# Risk factors associated with food consumption and food-handling habits for sporadic listeriosis: a case–control study in China from 2013 to 2022

**DOI:** 10.1080/22221751.2024.2307520

**Published:** 2024-02-11

**Authors:** Yanlin Niu, Weiwei Li, Biyao Xu, Wen Chen, Xiaojuan Qi, Yijing Zhou, Ping Fu, Xiaochen Ma, Yunchang Guo

**Affiliations:** a Beijing Center for Disease Prevention and Control, Beijing, People’s Republic of China; b National Health Commission Key Laboratory of Food Safety Risk Assessment, Chinese Academy of Medical Science Research Unit (No.2019RU014), China National Center for Food Safety Risk Assessment, Beijing, People’s Republic of China; c Shanghai Municipal Center for Disease Control and Prevention, Shanghai, People’s Republic of China; d Sichuan Center for Disease Control and Prevention, Chengdu, People’s Republic of China; e Zhejiang Provincial Center for Disease Control and Prevention, Hangzhou, People’s Republic of China; f Jiangsu Provincial Center for Disease Control and Prevention, Nanjing, People’s Republic of China

**Keywords:** case–control study, listeriosis, risk factor, food consumption, food-handling habit, perinatal

## Abstract

The prevalence of listeriosis in China has been increasing in recent years. Listeriosis primarily spreads through contaminated food. However, the resilient causative organism, *Listeria monocytogenes*, and its extended incubation period pose challenges in identifying risk factors associated with food consumption and food-handling habits. This study aimed to identify the risk factors associated with food consumption and food-handling habits for listeriosis in China. A matched case–control study (1:1 ratio) was conducted, which enrolled all eligible cases of listeriosis between 1 January 2013 and 31 December 2022 in China. Basic information and possible risk factors associated with food consumption and food-handling habits were collected. Overall, 359 patients were enrolled, including 208 perinatal and 151 non-perinatal cases. Univariate and multivariable logistic analyzes were performed for the perinatal group. For the perinatal and non-perinatal groups, ice cream and Chinese cold dishes were the high-risk foods for listeriosis (odds ratio (OR) 2.09 95% confidence interval (CI): 1.23–3.55; OR 3.17 95% CI: 1.29–7.81), respectively; consumption of leftovers and pet ownership were the high-risk food-handling habits (OR 1.92 95% CI: 1.03–3.59; OR 3.00 95% CI: 1.11–8.11), respectively. In both groups, separation of raw and cooked foods was a protective factor (OR 0.27 95% CI: 0.14–0.51; OR 0.35 95% CI: 0.14–0.89), while refrigerator cleaning reduced the infection risk by 64.94–70.41% only in the perinatal group. The identification of high-risk foods and food-handling habits for listeriosis is important for improving food safety guidelines for vulnerable populations.

## Introduction

*Listeria monocytogenes* is a gram-positive facultative intracellular pathogen that causes human listeriosis; it is ubiquitous in the environment and replicates at low temperatures and a wide pH range [1,2]. Listeriosis can manifest across a spectrum of clinical presentations, including febrile gastroenteritis, septicaemia, and central nervous system (CNS) infections, which can be fatal in older adults, neonates, and immunocompromised individuals. Listeriosis can also result in septic abortion or stillbirth in pregnant women [3,4].

Globally, the annual incidence of listeriosis is estimated to be approximately three to six cases per 1 million people, with mortality rates ranging from 20 to 40% [3,5]. In China, 147 sporadic cases and 82 outbreak cases of listeriosis were reported between 1964 and 2010, with mortality rates of 26% for overall listeriosis and 46% for neonatal listeriosis [6]. A systematic review showed 562 patients were diagnosed with listeriosis in China between 2011 and 2017; this number was much higher than that reported in the previous decade, with a mortality rate of 23.78% in non-perinatal patients and 32.68% of perinatal patients resulting in abortion and/or newborn death [7]. The burden of listeriosis in China may have been underestimated due to the absence of a comprehensive Listeria infection monitoring system.

Listeriosis primarily spreads through contaminated food [8]. However, the ubiquitous and cold-tolerant nature of *L. monocytogenes* and its long and variable incubation period pose challenges in identifying risk factors associated with food consumption and food-handling habits [9,10]. In 1981, cabbage was first identified as the source of infection in an investigation of an outbreak in Nova Scotia, Canada [11]. Subsequent outbreaks have been linked to contaminants present in milk, soft cheese, ready-to-eat meat, and other food sources [12–16]. No outbreaks have been recorded in China before 1981, making it difficult to identify relevant food sources [7].

Food consumption patterns and food handling habits in China notably differ from those in Western countries, which renders it inappropriate to directly extrapolate conclusions regarding the risk factors associated with listeriosis infection to Western studies. In our previous study conducted in Beijing, China, we found that cold Chinese dishes and the lack of separation of raw and cooked food may be risk factors for *L. monocytogenes* infection in non-perinatal and perinatal patients, respectively [17]. However, given the limited sample size and geographical range, further studies are required to validate the generalisability of these findings. Therefore, this national case–control study aimed to identify the risk factors associated with food consumption and food-handling habits in sporadic listeriosis in China. This study covered a number of important regions and collected data on cases in China over a decade.

## Methods

### Surveillance of listeriosis in China

In 2013, a special surveillance initiative for listeriosis was launched and subsequently integrated into the National Foodborne Disease Surveillance Plan in China. Medical institutions with the capacity to diagnose and treat foodborne diseases are required to report listeriosis cases via the National Foodborne Disease Surveillance System [18]. Listeriosis was classified as a notifiable foodborne disease according to the requirements of the Foodborne Disease Surveillance and Reporting Specifications issued by the National Health Commission in 2019 [19].

For surveillance, listeriosis cases were defined as patients from whom *L. monocytogenes* was isolated from a sterile site such as blood or cerebrospinal fluid (CSF) through blood cultures or other culture methods in a clinical laboratory, besides the presence of symptoms such as fever, bacteraemia, sepsis, or other clinical manifestations corresponding to listeriosis. These cases were further classified into non-perinatal cases (patients who were not pregnant women or newborns) and perinatal cases (patients who were pregnant women or newborns, and cases of foetal loss). Cases in which both the mother and newborn, or foetus, were positive for *L. monocytogenes* were counted as a single case. To identify the possible outbreaks, whole-genome sequencing method was used for the homology analysis.

### Design of case–control study

To identify the risk factors for listeriosis, particularly those associated with food consumption and food-handling habits, we conducted a matched case–control study (1:1 ratio) similar to our previous study [17]. We enrolled all listeriosis cases in China from 1 January 2013–31 December 2022. For all perinatal cases, including those in which only the infant was clinically ill, the mother was considered the case patient for recording the information required in this study. Cases were excluded if they met any of the following criteria: (1) were asymptomatic, except for perinatal mothers; (2) part of an outbreak associated with an identified food vehicle, identified by whole-genome sequencing and epidemiological investigation; (3) could not be contacted within 4 weeks of the culture date; and (4) when the treating doctor or family member refused participation. Controls were selected from the same hospital as the matched cases. General matching conditions included sex and age within 5 years. Additionally, for non-perinatal cases, controls were required to share similar underlying immune conditions with the matched cases. Specifically, the case–control pairs with hypoimmunity were required to have similar causes, such as organ transplantation, tumours, or renal disease, to minimize the influence of possible changes in eating habits caused by a particular disease. For the perinatal cases, the gestational age of the controls was within a 2-week range. All controls were recruited within 4 weeks of conducting the case interviews. Patients who could not be matched with controls were excluded.

Face-to-face interviews were conducted with patients or their surrogates and controls by staff of the Centers for Disease Control and Prevention (CDC) using a standard structured questionnaire. Basic patient information and possible risk factors associated with food consumption and food-handling habits were obtained from the patients or surrogates. Combined with dietary habits with Chinese characteristics, this study investigated the consumption of 8 food items identified as having a high risk of *Listeria* contamination in a previous study [2]. Different from Western food, Chinese food is known for its variety, complexity, and unique flavours. As one of the most representative Chinese foods, cold dishes may usually be prepared from cooked and/or raw ingredients in advance, stored at room temperature before serving and not generally reheated before consumption [17]. The investigation covered the 4 weeks preceding the specimen collection date for cases, the corresponding 4 weeks for perinatal controls, and the 4 weeks preceding the interview date for non-perinatal controls. Moreover, this study investigated eight types of food-handling habits, including handling of poultry meat, the separation of raw and cooked foods, frequency of refrigerator cleaning, consumption of leftovers, heating leftovers before consumption, banquet attendance, history of contact with live poultry and pet ownership. This study was approved by the Ethics Committee of the China National Center for Food Safety Risk Assessment. Written informed consent was obtained from all patients and controls before enrolment.

### Statistical analysis

**Figure 1.**
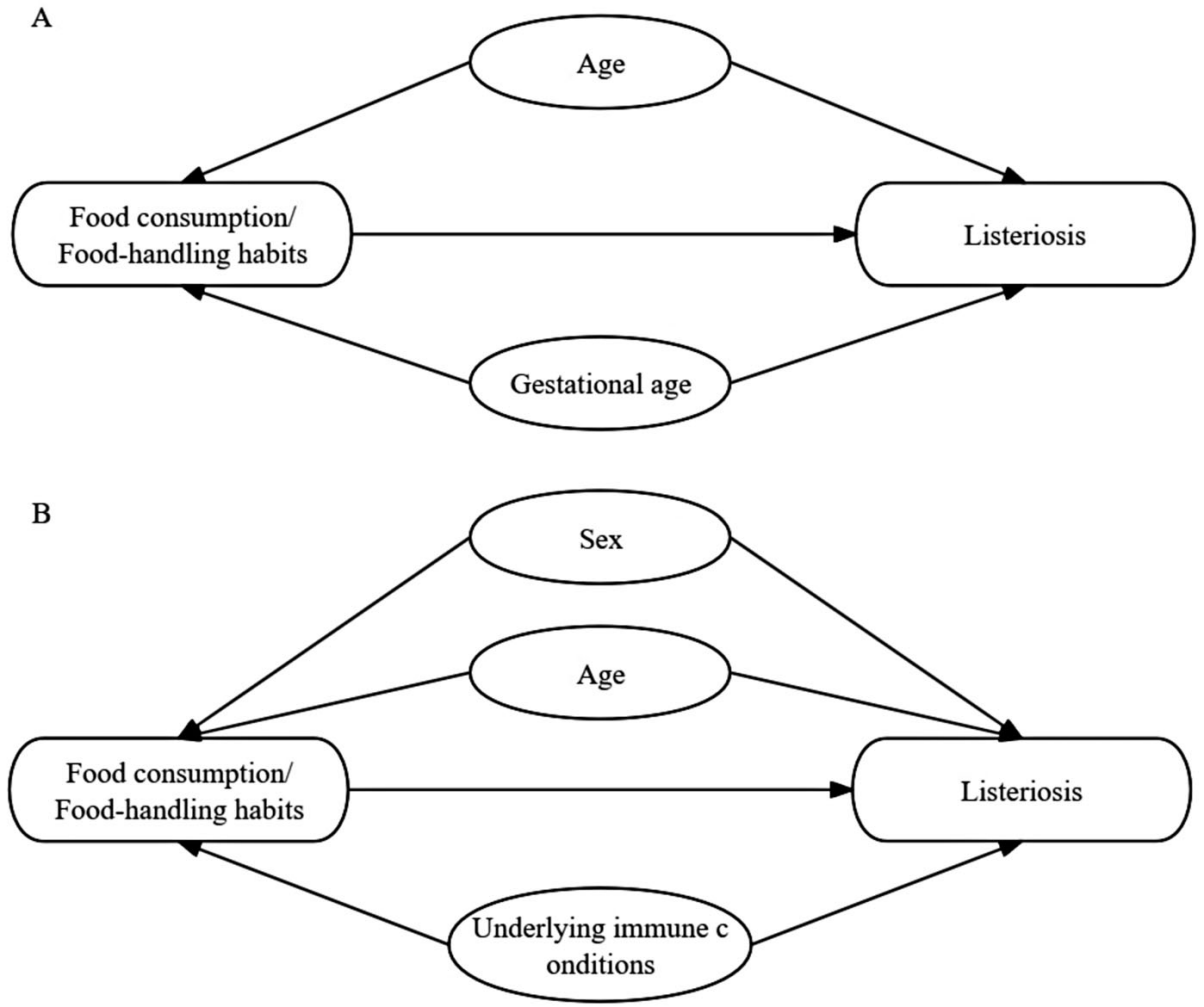
Directed acyclic graph (DAG) for the association between food consumption/food-handling habits and listeriosis in perinatal cases (A) and non-perinatal cases (B).

Descriptive analyzes were conducted for both perinatal and non-perinatal cases, as well as for controls. Univariate analysis was performed for all variables related to food consumption and food habits. Univariate odds ratios (ORs) and 95% confidence intervals (CIs) were computed using logistic regression adjusted for matching factors. Multivariable logistic regression modelling was used to investigate the independent relationships between risk factors and listeriosis while adjusting for the potential confounding effects of other factors. A forward stepwise selection strategy was employed, and all variables in the univariate analysis were included in the multivariable logistic analysis. Owing to the limited sample size of this study, food consumption and food-handling habits were analyzed in separate models instead of a combined model to obtain robust estimates. Directed acyclic graph (DAG) was used to identify the presence of confounding for causal assumptions between food consumption/food-handling habits and listeriosis (Figure 1). Statistical significance was set at *P* < 0.05. All reported *P*-values were two-tailed. All statistical analyzes were conducted using the “survival” packages in R (version 3.6.3).

## Results

### Basic information

**Figure 2.**
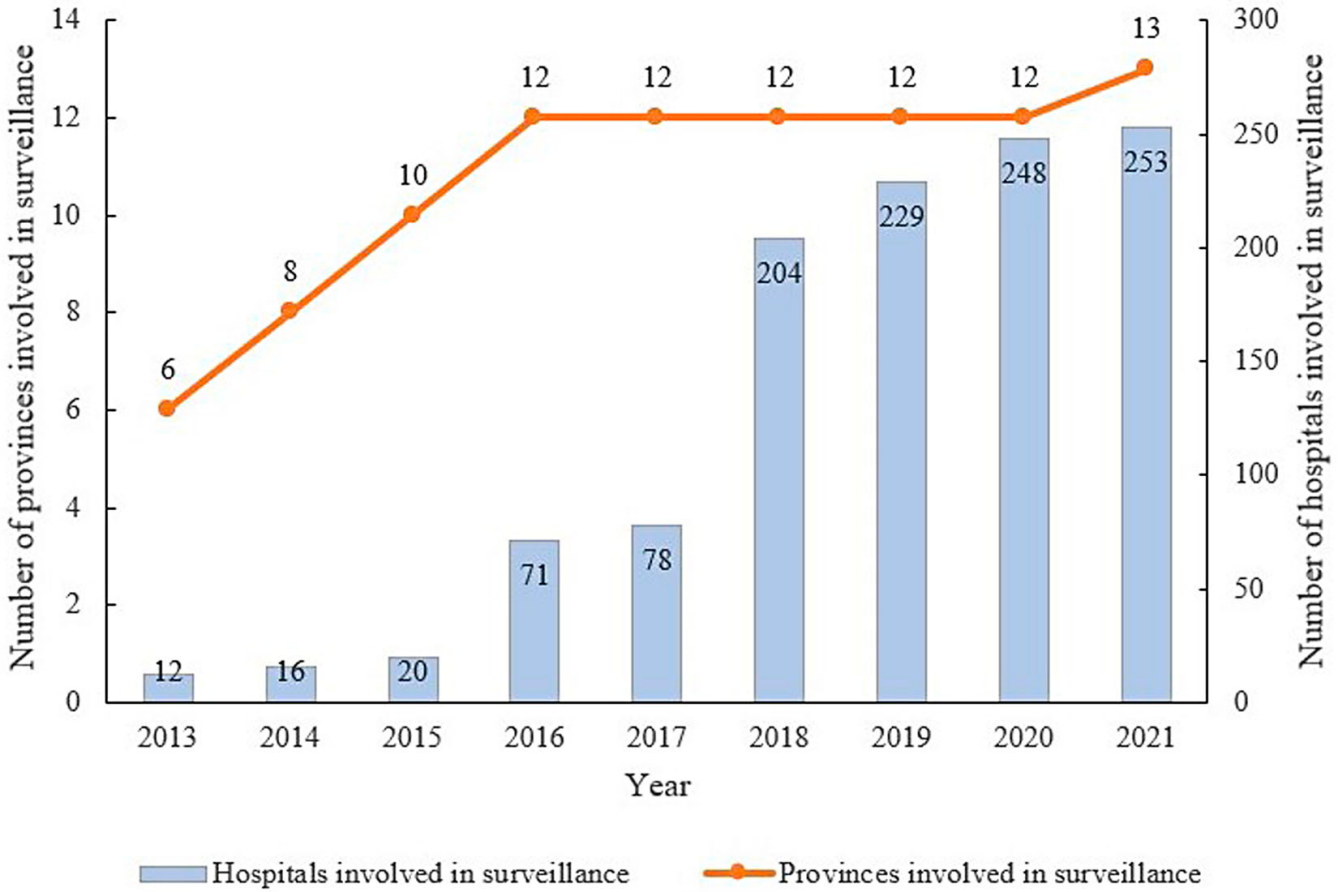
Progress of special surveillance for listeriosis in China.

**Figure 3.**
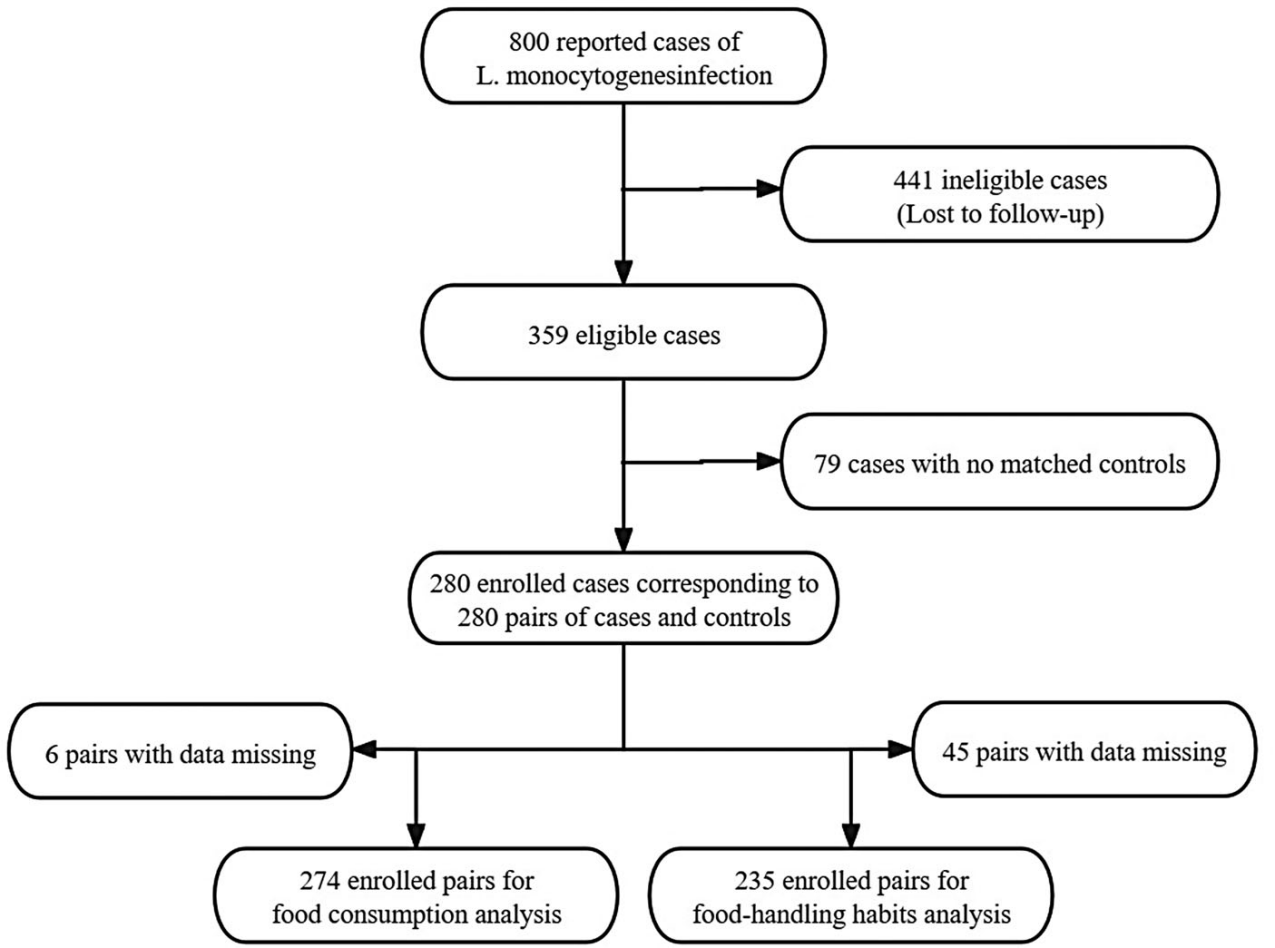
Eligibility and enrollment of patients with cases of *L. monocytogenes* in China from 2013 to 2022.

The initial phase of special surveillance for listeriosis in China initiated in 2013, covered 6 provinces and 12 hospitals across the country. By 2021, the programme had expanded to include 13 provinces and 253 hospitals. Since 2022, surveillance has covered all provinces in the country, with at least five sentinel hospitals selected in each province and required to report cases (Figure 2). In total, 800 cases were reported between 2013 and 2022, including 466 perinatal and 334 non-perinatal cases, and 66.63% (533/800) of clinical isolates were recovered and sequenced. Combined with whole-genome sequencing for available patient strains and epidemiological investigation of the cases, no outbreaks were identified during the study period. In the early stages of the project, the work process was not yet perfected. The lack of public education led to an insufficient understanding of listeriosis among patients, which led to a low degree of cooperation with the strain acquisition and the detailed investigation. In this study, 441 patients were lost to follow-up. Therefore, 359 cases were eligible, resulting in a response rate of 44.88% (359/800), including 208 perinatal and 151 non-perinatal cases. However, owing to the absence of matched controls, 79 cases, including 32 perinatal cases and 47 non-perinatal cases, were excluded. Finally, 280 cases, including 176 perinatal and 104 non-perinatal cases, were enrolled (Table 1, SM Table 1). The enrolled patients consisted of 280 pairs of cases and controls. After excluding missing data for key factors, 274 and 235 pairs were included in the analysis of food consumption and food-handling habits, respectively (Figure 3). Based on the mother's age, the range and the median of age in the perinatal group were 19.57–49.00 and 29.61 years, respectively, compared to 0.85–90.51 and 63.71 years in the non-perinatal group, respectively. The non-perinatal group comprised 56 males and 48 females.

**Table 1.** Basic information on listeriosis cases from 2013 to 2022 in China.

Items	Perinatal	Non-perinatal	Total
Reported cases	466	334	800
Eligible cases	208	151	359
Ineligible cases	240	165	405
Response rate	44.64%	45.21%	44.88%
No matched controls	32	47	79
Enrolled cases	176	104	280

### Univariate analysis

Univariate analysis showed that ice cream was a high-risk food for *L. monocytogenes* infection in the perinatal group, with an OR of 1.37 (95% *CI*: 1.08–1.73). However, in the non-perinatal group, no food-related risk factors were identified (Table 2).

**Table 2.** Food-related risk factors for sporadic listeriosis in China.

Consumption	Perinatal				Non-perinatal			
	Case(*n* = 174)	Control(*n* = 174)	OR (95% *CI*)		Case(*n* = 100)	Control(*n* = 100)	OR (95% *CI*)	
			Univariate	Multivariable			Univariate	Multivariable
Cooked meat products	103 (59.2)	112 (64.4)	0.88 (0.70–1.11)	0.79 (0.48–1.30)	62 (62.0)	56 (56.0)	1.15 (0.85–1.56)	1.12 (0.57–2.21)
Raw vegetables	96 (55.2)	95 (54.6)	1.01 (0.81–1.27)	1.02 (0.63–1.66)	45 (45.0)	48 (48.0)	0.93 (0.68–1.26)	0.69 (0.32–1.46)
Fruit	168 (96.6)	171 (98.3)	0.72 (0.37–1.38)	0.36 (0.08–1.54)	88 (88.0)	94 (94.0)	0.72 (0.45–1.13)	0.51 (0.18–1.44)
Freshly prepared drinks	36 (20.7)	47 (27.0)	0.85 (0.67–1.08)	0.69 (0.41–1.19)	10 (10.0)	10 (10.0)	1.00 (0.61–1.64)	1.07 (0.36–3.17)
Ice cream	85 (48.9)	64 (36.8)	**1.37** (**1.08–1.73)**	**2.09** (**1.23–3.55)**	18 (18.0)	14 (14.0)	1.18 (0.79–1.76)	1.55 (0.62–3.87)
Chinese cold dishes	107 (61.5)	105 (60.3)	1.03 (0.81–1.31)	1.11 (0.67–1.86)	49 (49.0)	40 (40.0)	1.20 (0.91–1.59)	**1.94** (**0.96–3.94)**
Western-style salad	28 (16.1)	31 (17.8)	0.96 (0.73–1.27)	1.08 (0.58–1.99)	3 (3.0)	5 (5.0)	0.72 (0.32–1.60)	0.40 (0.06–2.59)
Cheese	33 (19.0)	39 (22.4)	0.89 (0.68–1.17)	0.85 (0.46–1.56)	10 (10.0)	8 (8.0)	1.13 (0.69–1.85)	1.28 (0.44–3.73)

Note: OR, odds ratio; CI, confidence interval

Regarding dietary habits, separation of raw and cooked foods appeared to be a common protective factor against *L. monocytogenes* infection in both the perinatal and non-perinatal groups, with ORs of 0.57 (95% *CI*: 0.46–0.71) and 0.62 (95% *CI*: 0.45–0.86), respectively. Additionally, refrigerator cleaning was identified as a protective factor in the perinatal group, with ORs ranging from 0.54–0.59. However, the protective effect did not show an evident upward trend with increasing frequency. On the contrary, leftover consumption was identified as a risk factor in the perinatal group, which increased the risk of infection by 39.56% (95% *CI*: 9.57% – 77.76%).% In the non-perinatal group, the risk of *L. monocytogenes* infection was influenced by handling poultry meat and pet ownership. Both were risk factors for listeriosis, with ORs of 1.42 (95% *CI*: 1.02–1.98) and 1.59 (95% *CI*: 1.09–2.31), respectively (Table 3).

**Table 3.** Risk factors associated with living habits for sporadic listeriosis in China.

Dietary habits	Perinatal				Non-perinatal			
	Case(*n* = 151)	Control(*n* = 151)	OR (95% CI)		Case(*n* = 84)	Control(*n* = 84)	OR (95% CI)	
			Univariate	Multivariable			Univariate	Multivariable
Handling of poultrymeat (Yes)^a^	92 (60.9)	88 (58.3)	1.07 (0.83–1.39)	1.06 (0.56–2.00)	55 (65.5)	43 (51.2)	**1.42(1.02–1.98)**	1.87 (0.84–4.18)
Separation of raw andcooked foods (Yes) ^a^	66 (43.7)	104 (68.9)	**0.57** (**0.46–0.71)**	**0.27** (**0.14–0.51)**	45 (53.6)	61 (72.6)	**0.62** (**0.45–0.86)**	**0.35** (**0.14–0.89)**
Frequency ofrefrigerator cleaning								
<1 time/year^b^	66 (43.7)	32 (21.2)	–	–	28 (33.3)	24 (28.6)	–	–
<1–2 times/year	49 (32.5)	65 (43.0)	**0.59** (**0.46–0.77)**	**0.30** (**0.15–0.59)**	32 (38.1)	34 (40.5)	0.88 (0.61–1.25)	0.97 (0.41–2.31)
<3–5 times/year	19 (12.6)	29 (19.2)	**0.54** (**0.39–0.76)**	**0.34** (**0.14–0.82)**	15 (17.9)	11 (13.1)	1.12 (0.69–1.80)	1.32 (0.43–4.10)
>5 times/year	17 (11.3)	25 (16.6)	**0.57** (**0.40–0.80)**	**0.35** (**0.13–0.91)**	9 (10.7)	15 (17.9)	0.66 (0.39–1.11)	0.56 (0.15–2.05)
Consumption ofleftovers (Yes) ^a^	86 (57.0)	65 (43.0)	**1.40** (**1.10–1.78)**	**1.92** (**1.03–3.59)**	53 (63.1)	54 (64.3)	0.97 (0.69–1.37)	0.60 (0.25–1.42)
Heating leftoversbefore consumption								
Almost always	63 (73.3)	50 (76.9)	0.60 (0.19–1.86)	–	32 (60.4)	38 (70.4)	–	–
Often	9 (10.5)	6 (9.2)	0.79 (0.17–3.58)	–	12 (22.6)	7 (13.0)	–	–
Occasionally^b^	14 (16.3)	9 (13.8)	–	–	9 (17.0)	9 (16.7)	–	–
Banquet attendance(Yes) ^a^	33 (21.9)	23 (15.2)	1.27 (0.94–1.71)	1.62 (0.80–3.27)	18 (21.4)	13 (15.5)	1.24 (0.83–1.87)	1.39 (0.52–3.67)
History of contact withlive poultry (Yes) ^a^	5 (3.3)	5 (3.3)	1.00 (0.50–2.00)	0.78 (0.14–4.35)	12 (14.3)	7 (8.3)	1.34 (0.84–2.16)	0.83 (0.24–2.87)
Pet ownership (Yes) ^a^	14 (9.3)	18 (11.9)	0.88 (0.61–1.25)	0.76 (0.31–1.87)	22 (26.2)	10 (11.9)	**1.59** (**1.09–2.31)**	**3.00** (**1.11–8.11)**

Note: OR, odds ratio; CI, confidence interval.

^a^ Take No as the reference group.

^b^ Reference group in the model.

### Multivariable analysis

The results of the multivariable analysis showed that ice cream was a high-risk food for *L. monocytogenes* infection in the perinatal group, which increased the risk by 2.09-fold (95% *CI*: 1.23–3.55). For the non-perinatal group, Chinese cold dishes seemed to be a high-risk food with an OR of 1.94 (95% *CI*: 0.96–3.94) (Table 2). Due to differences in dietary patterns between North and South China, Chinese cold dishes are rarely eaten in South China. Therefore, a multivariable stratified analysis by north and south was further carried out in the non-perinatal group, which showed that Chinese cold dishes increased the risk of infection by 3.17-fold (95% *CI*: 1.29 - 7.81) in the north of China. However, the model for southern China did not converge, and the results were not robust (SM Table 2).

Regarding dietary habits, the results of the multivariable analysis were similar to those of the univariate analysis. The practice of separating raw and cooked foods remained a common and effective protective factor against listeriosis in both the perinatal and non-perinatal groups, with ORs of 0.27 (95% *CI*: 0.14–0.51) and 0.35 (95% *CI*: 0.14–0.89), respectively. Additionally, refrigerator cleaning was identified as a protective factor in the perinatal group, reducing the infection risk by 64.94–70.41%, compared with refrigerator cleaning less than once a year. Regarding risk factors, the consumption of leftovers increased the risk of infection by 92.48% (95% *CI*: 3.30% – 258.64%) in the perinatal group. Furthermore, a specific univariate analysis was conducted targeting the frequency of heating leftovers before consumption in the perinatal group. However, whether the leftovers were heated before consumption showed no significant difference in the infection risk. In the non-perinatal group, pet ownership was identified as a risk factor, increasing the infection risk by up to 3-fold (95% *CI*: 1.11–8.11) (Table 3).

## Discussion

This is the first national case–control study of sporadic listeriosis in China, which aimed to identify the factors associated with food consumption and food-handling habits specific to Chinese dietary habits. The results showed that ice cream and Chinese cold dishes were high-risk foods for *L. monocytogenes* infection in the perinatal and non-perinatal groups, respectively. Risk factors associated with food-handling habits included the consumption of leftovers and pet ownership in the perinatal and non-perinatal groups, respectively. The habit of separating raw and cooked foods was found to be a protective factor against the *L. monocytogenes* infection in both the perinatal and non-perinatal groups. Refrigerator cleaning was identified as another protective factor associated with food-handling habits in the perinatal group. Our findings provide a scientific and accurate basis for the formulation of prevention and control measures for listeriosis to greatly benefit vulnerable populations at high risk.

This study identified ice cream as a high-risk food for listeriosis in the perinatal group. From the conventional viewpoint, ice cream is considered to have a low risk of transmission of *L. monocytogenes* as the level of contamination is usually low, and the freezing temperature can prevent pathogen growth [20]. However, ice cream has been linked to an outbreak of *L. monocytogenes* in the US [21–23]. As no such outbreaks have been identified in China, no high-risk foods, including ice cream, have been identified. A study from China showed that the positive rate of *L. monocytogenes* in ice cream was 0.62%, and the loads for positive samples were in the range of 0.3–240 most probable number (MPN)/g with only one sample >100 MPN/g, indicating a relatively low contamination level [24]. Similarly, an extended investigation of the US outbreak found a high prevalence of *L. monocytogenes* but at very low loads in ice cream [25]. This indicates that the majority of the population may not become infected after ingesting low doses of *L. monocytogenes* when no growth is facilitated; however, the illness could occur in the highly susceptible population after the distribution of low-level contaminated products that do not support the growth of this pathogen [22]. This also suggests that the underlying health of a patient, immune status, and the medication that they take may play a more crucial role than the dose [26]. Moreover, irregularities in ice cream processing, transportation, and storage, such as insufficient cooling at street vendors’ places and the use of thawed ingredients, can also pose risks and should not be ignored. Considering the high risk of *L. monocytogenes* infection in susceptible groups, stringent control on the limits of *L. monocytogenes* as a pathogenic bacterial indicator in food is essential. *L. monocytogenes* was added as a microbial index (not detected) to ice cream for the first time in the new Chinese national standards issued in September 2021 [27,28]. Consistent with a previous study conducted in Beijing, China [17], this study found that cold Chinese dishes were high-risk foods for *L. monocytogenes* infection in the non-perinatal group, especially in northern China. Cold dishes are more commonly consumed in northern China, especially in the summer. Most Chinese cold dishes are manufactured from raw vegetables and cooked meat and are not heated during manufacturing or before consumption. One common aspect of such foods with ready-to-eat foods is that they are consumed without further listericidal steps, which increases the risk of *L. monocytogenes* infection through contaminated food. A study on the prevalence of *L. monocytogenes* in food products in China reported that *L. monocytogenes* was found in 3.65% of the sampled Chinese cold dishes [29]. The prevalence of *L. monocytogenes* in vegetables and cooked meat, which were the major ingredients of Chinese cold dishes, were 2.84% and 4.61%, respectively. The high level of *L. monocytogenes* in Chinese cold dishes and their ingredients presented in the above data supported our conclusions.

This study found that consumption of leftovers was a risk factor in the perinatal group. A simulation study of pork meat found that a few surviving cells of *L. monocytogenes* could multiply during storage after cooking treatments [30]. Although reheating can somewhat reduce bacterial counts, many *L. monocytogenes* survivors can still be found after reheating, especially after extended storage periods [31]. This may explain our finding that heating leftovers before consumption did not reduce the risk of infection. However, reheating the leftovers until steaming is highly recommended for pregnant women to prevent listeriosis. Notably, storage practices, including the probability of pathogen occurrence after cooking, degree of doneness, duration of storage, and duration of storage at room temperature, were the key factors affecting the exposure of consumers to *L. monocytogenes*, and awareness of these factors is essential [30]. However, we did not find that the consumption of leftovers could increase the infection risk in the non-perinatal group. We speculate that pregnant women in a special physiological period may be more sensitive to bacteria in leftovers. The relatively small sample size of the non-pregnant group may also result in a low power to detect the influencing factors. Besides, pet ownership was identified as a risk factor in the non-perinatal group. Pets, often asymptomatic carriers of pathogens, including *L. monocytogenes*, can excrete them via their faeces, thus delivering them to the environment. Consequently, these pathogens may infect new individuals and end up on vegetables and fruits [32]. Notably, few pregnant women keep pets to ensure the safety of perinatal mothers and children due to the high prevalence of pathogens in pets. This may explain why pet ownership was not a risk factor in the perinatal group. Moreover, foodborne pathogen outbreaks associated with pet food have highlighted these products as vehicles for pathogens in both pets and their owners [33]. One study showed that presumptive *Listeria* species were detected in 64% of pet food product brands in Lebanon [34]. An investigation also reported that more than 6% of respondents acknowledged incidents of involuntary ingestion of pet snacks, resulting in fever and gastrointestinal manifestations. In the cited study, children and infants are particularly vulnerable, as they are more likely to come into contact with pet snacks due to their toy-like appearance [33]. The upward trend in pet ownership and close contact between people and pets may also increase the likelihood of infection.

Regarding dietary habits, the separation of raw and cooked foods was identified as a protective factor in both the perinatal and non-perinatal groups, consistent with the observations of our previous study [17]. Listeria species are commonly found in raw and unprocessed food products and serve as key vehicles for listeriosis [12,35]. The prevalence of *L. monocytogenes* in raw meat and poultry in China can exceed 11%, which is significantly higher than that in cooked meat (4.61%) [29]. Mixing raw and cooked foods during processing and storage can lead to contamination by *L. monocytogenes*, greatly increasing the risk of infection. In addition, refrigerator cleaning was identified as another protective factor associated with food-handling habits in the perinatal group. Domestic refrigerators are potential sources of food pathogen contamination. Bacteria from unwashed raw food products, leaking packages, unclean hands, and unclean container surfaces introduced into domestic refrigerators can directly contaminate other stored foods and persist on the internal refrigerator surfaces. This, in turn, increases the risk of long-term indirect contamination during food preparation [36]. *L. monocytogenes* has been isolated from up to 60% of refrigerator areas [37]. Furthermore, a weak correlation was found between refrigerator cleaning and the presence of bacteria, emphasizing the importance of frequent cleaning to reduce contamination in domestic refrigerators [38,39]. Although it may not be possible to completely eliminate food pathogens from refrigerators, their spread, growth, and survival can be controlled using the correct cleaning frequency and methods [39]. Similar to the consumption of leftovers, refrigerator cleaning was not identified in the non-perinatal group, which may also be caused by the high sensitivity in the perinatal group and the small sample size in the non-perinatal group.

Our study has a few limitations. First, there was selection bias. Some patients were excluded from this study because they died or were too ill to respond to the questions. Families who experienced adverse outcomes, such as death or stillbirth, often refused to participate in the study because of traumatic stress. The excluded patients may represent a population with unique host dynamics or food exposure. Additionally, the recruitment of control participants for this study was challenging because no biological or epidemiological methods exist to measure and adjust for susceptibility to listeriosis. We had to rely on physician practice for recruitment, as in the study by Varma et al. [40], which may have introduced bias. However, the cases and controls were matched according to demographic information and medical conditions to minimize bias. Second, there is a possibility of information bias. Given the long exposure period for dietary histories, the interviewees were required to recall their food consumption from more than 4 weeks earlier, which may have led to a higher likelihood of reporting their commonly eaten foods rather than their exact exposures. Moreover, surrogates for unavailable patients may have led to misclassification of exposure. Third, confounding bias cannot be completely controlled. The limited sample size made it hard to develop an integrated model incorporating food consumption and food-handling habits, resulting in the possible cross-confounding bias. Finally, the socioeconomic status of the cases and controls, an important confounding factor, could not be assessed and included in the model, which may have influenced the results.

## Conclusion

Ice cream and Chinese cold dishes have been identified as risk foods for *L. monocytogenes* infection. Consumption of leftovers and close contact with pets should be avoided, as much as possible, by the at-risk population. The habit of separating raw and cooked foods and refrigerator cleaning proved to be effective ways to reduce the risk of *L. monocytogenes* infection. As a primary foodborne pathogen with a potentially underestimated disease burden in China, *L. monocytogenes* has recently received increased attention from the public due to the increasing morbidity and mortality in vulnerable populations. Identifying risk factors associated with food consumption and food-handling habits in sporadic listeriosis is valuable for improving food safety guidelines, especially for pregnant women and immunocompromised individuals.

## Supplementary Material

Supplementary_Materials_without_Change_Track

Sample_Size_Report

Certificate_of_editing
