# Supplementary material for: Risk factors associated with food consumption and food-handling habits for sporadic listeriosis: a case–control study in China from 2013 to 2022
Source: Emerg Microbes Infect. 2024 Feb 11;13(1):2307520. doi: 10.1080/22221751.2024.2307520 (PMC10860432; doi:10.1080/22221751.2024.2307520)
Supplement: Supplementary_Materials_without_Change_Track [file TEMI_A_2307520_SM2883.docx]

SM Table 1. Yearly basic information on listeriosis cases from 2013 to 2022 in China

| Year/Type | Reported cases | Eligible cases | Ineligible cases | Response rate | No matched controls | Enrolled cases |
| --- | --- | --- | --- | --- | --- | --- |
| 2013 | 2 | 0 | 2 | 0 | 0 | 0 |
| Perinatal | 2 | 0 | 2 | 0 | 0 | 0 |
| Non-perinatal | 0 | 0 | 0 | - | 0 | 0 |
| 2014 | 21 | 9 | 14 | 42.86% | 6 | 3 |
| Perinatal | 17 | 7 | 9 | 41.18% | 4 | 3 |
| Non-perinatal | 4 | 2 | 5 | 50.00% | 2 | 0 |
| 2015 | 37 | 17 | 20 | 45.95% | 13 | 4 |
| Perinatal | 17 | 6 | 11 | 35.29% | 2 | 4 |
| Non-perinatal | 20 | 11 | 9 | 55.00% | 11 | 0 |
| 2016 | 83 | 25 | 56 | 30.12% | 14 | 11 |
| Perinatal | 58 | 14 | 42 | 24.14% | 3 | 11 |
| Non-perinatal | 25 | 11 | 14 | 44.00% | 11 | 0 |
| 2017 | 84 | 31 | 54 | 36.90% | 14 | 17 |
| Perinatal | 54 | 19 | 36 | 35.19% | 2 | 17 |
| Non-perinatal | 30 | 12 | 18 | 40.00% | 12 | 0 |
| 2018 | 102 | 54 | 48 | 52.94% | 4 | 50 |
| Perinatal | 50 | 28 | 20 | 56.00% | 1 | 27 |
| Non-perinatal | 52 | 26 | 28 | 50.00% | 3 | 23 |
| 2019 | 143 | 83 | 66 | 58.04% | 3 | 80 |
| Perinatal | 102 | 56 | 49 | 54.90% | 3 | 53 |
| Non-perinatal | 41 | 27 | 17 | 65.85% | 0 | 27 |
| 2020 | 98 | 40 | 61 | 40.82% | 11 | 29 |
| Perinatal | 55 | 24 | 33 | 43.64% | 8 | 16 |
| Non-perinatal | 43 | 16 | 28 | 37.21% | 3 | 13 |
| 2021 | 121 | 63 | 58 | 52.07% | 8 | 55 |
| Perinatal | 62 | 36 | 26 | 58.06% | 5 | 31 |
| Non-perinatal | 59 | 27 | 32 | 45.76% | 3 | 24 |
| 2022 | 109 | 37 | 26 | 33.94% | 6 | 31 |
| Perinatal | 49 | 18 | 12 | 36.73% | 4 | 14 |
| Non-perinatal | 60 | 19 | 14 | 31.67% | 2 | 17 |

SM Table 2. Food-related risk factors for non-perinatal listeriosis in north and south of China

| Consumption | The North | | | The South^a^ | | |
| --- | --- | --- | --- | --- | --- | --- |
|  | Case (n=75) | Control (n=75) | OR (95% *CI*) | Case (n=25) | Control (n=25) | OR (95% *CI*) |
| Cooked meat products | 50 (66.7) | 46 (61.3) | 0.81(0.32-2.00) | 12 (48.0) | 10 (40.0) | 5.48(1.23-24.37) |
| Raw vegetables | 41 (54.7) | 40 (53.3) | 0.74(0.28-1.91) | 4 (16.0) | 8 (32.0) | 0.13(0.01-1.57) |
| Fruit | 65 (86.7) | 71 (94.7) | 0.33(0.09-1.22) | 23 (92.0) | 23 (92.0) | 1.27(0.10-15.56) |
| Freshly prepared drinks | 9 (12.0) | 7 (9.3) | 1.68(0.45-6.24) | 1 (4.0) | 3 (12.0) | 0.00(0.00-Inf) |
| Ice cream | 14 (18.7) | 8 (10.7) | 2.45(0.78-7.66) | 4 (16.0) | 6 (24.0) | 0.00(0.00-Inf) |
| Chinese cold dishes | 42 (56.0) | 31 (41.3) | **3.17(1.29-7.81)** | 7 (28.0) | 9 (36.0) | 0.26(0.03-1.96) |
| Western-style salad | 2 (2.7) | 5 (6.7) | 0.14(0.01-1.54) | 1 (4.0) | 0 (0.0) | 17418016588.21(0.00-Inf) |
| Cheese | 8 (10.7) | 7 (9.3) | 0.94(0.28-3.11) | 2 (8.0) | 1 (4.0) | 24541058811.98(0.00-Inf) |
| Note: OR, odds ratio; CI, confidence interval.  ^a^ Ran out of iterations and did not converge | | | | | | |
