# Supplementary material for: Risk factors associated with food consumption and food-handling habits for sporadic listeriosis: a case–control study in China from 2013 to 2022
Source: Emerg Microbes Infect. 2024 Feb 11;13(1):2307520. doi: 10.1080/22221751.2024.2307520 (PMC10860432; doi:10.1080/22221751.2024.2307520)
Supplement: Sample_Size_Report [file TEMI_A_2307520_SM2880.pdf]

Tests for the Odds Ratio in a Matched Case-Control Design with a Binary X

Numeric Results

Hypothesis Type: Two-Sided

Power	Number of Matched Sets N	Cases per Set M_D	Controls per Set M_H	Odds Ratio OR	Probability of Risk Exposure P_E	Regression of Exposure on other Covariates R^2	Alpha
0.90144	65	1	1	3.43	0.31	0.00	0.05
0.90291	66	1	1	3.43	0.31	0.01	0.05
0.90381	69	1	1	3.43	0.31	0.05	0.05
0.90057	72	1	1	3.43	0.31	0.10	0.05
0.90338	77	1	1	3.43	0.31	0.15	0.05
0.90057	81	1	1	3.43	0.31	0.20	0.05

References

Lachin, John M. 2008. 'Sample size evaluaion for a multiply matched case-control study using the score test from a conditional logistic (discrete Cox PH) regression model.' Statistics in Medicine, Volume 27, Pages 2509-2523.

Lachin, John M. 2011. Biostatistical Methods: The Assessment of Relative Risks, Second Edition. John Wiley & Sons. New York.

Tang, Yongqiang. 2009. 'Comments on ‘Sample size evaluation for multiply matched case-control study using the score test from a conditional logistic (discrete Cox PH) regression model.'' Statistics in Medicine, Volume 28, Pages 175-177.

Report Definitions

Power is the probability of rejecting a false null hypothesis.

Number of Matched Sets, N, is the number of sets (strata) in the study. Each set consists in a fixed number of cases and controls.

Cases per Set, M_D, is the number cases in each matched set.

Controls per Set, M_H, is the number of controls in each matched set.

Odds Ratio, OR, is the odds ratio of developing a disease associated with exposure to a certain risk factor.

Probability of Risk Exposure, P_E, is the probability of exposure to the risk factor in the overall population.

Regression of Exposure on other Covariates, R^2, is the R^2 that occurs when the exposure variable is regressed on any other covariates. This adjustment assumes that covariates that have a large correlation with the outcome are used in the matching process and are not included here.

Alpha is the probability of rejecting a true null hypothesis of no association between disease and the exposure variable.

Summary Statements

In a matched case-control study, a sample of 65 matched sets (or strata) is obtained. Each matched set consists of 1 case and 1 control. The probability of exposure to the risk factor in the population is 0.31. This sample achieves 90% power to detect an odds ratio of 3.43 calculated using conditional logistic regression with a 0.05 significance level.

Tests for the Odds Ratio in a Matched Case-Control Design with a Binary X

Dropout-Inflated Sample Size

Dropout Rate	Sample Size N	Dropout-Inflated Enrollment Sample Size N'	Expected Number of Dropouts D
20%	65	82	17
20%	66	83	17
20%	69	87	18
20%	72	90	18
20%	77	97	20
20%	81	102	21

Definitions

Dropout Rate (DR) is the percentage of subjects (or items) that are expected to be lost at random during the course of the study and for whom no response data will be collected (i.e., will be treated as "missing").

N is the evaluable sample size at which power is computed (as entered by the user). If N subjects are evaluated out of the N' subjects that are enrolled in the study, the design will achieve the stated power.

N' is the total number of subjects that should be enrolled in the study in order to end up with N evaluable subjects, based on the assumed dropout rate. N' is calculated by inflating N using the formula N' = N / (1 - DR), with N' always rounded up. (See Julious, S.A. (2010) pages 52-53, or Chow, S.C., Shao, J., Wang, H., and Lokhnygina, Y. (2018) pages 32-33.)

D is the expected number of dropouts. D = N' - N.

Chart Section

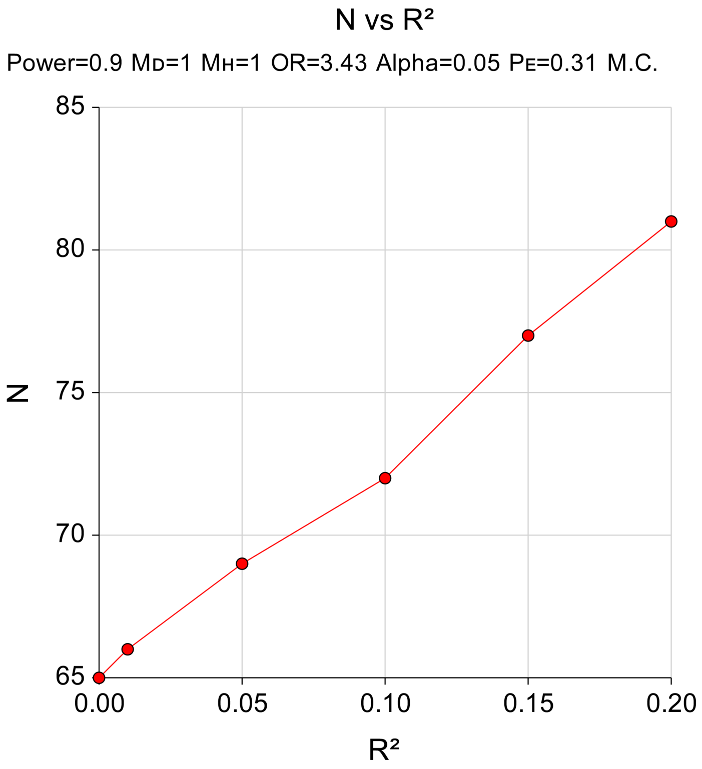

Tests for the Odds Ratio in a Matched Case-Control Design with a Binary X

Procedure Input Settings

Autosave Inactive

Design Tab

Solve For: Sample Size N
Alternative Hypothesis: Two-Sided
Power: 0.90
Alpha: 0.05
M (Number of Cases per Set): 1
Mн (Number of Controls per Set):
1
OR (Odds Ratio): 3.43
P (Probability of Exposure): 0.31
R^2 (Exposure vs. Covariates): 0 0.01 0.05 0.1 0.15 0.2
