# Supplementary material for: Risk factors associated with food consumption and food-handling habits for sporadic listeriosis: a case–control study in China from 2013 to 2022
Source: Emerg Microbes Infect. 2024 Feb 11;13(1):2307520. doi: 10.1080/22221751.2024.2307520 (PMC10860432; doi:10.1080/22221751.2024.2307520)
Supplement: Certificate_of_editing [file TEMI_A_2307520_SM2879.pdf]

editage

Editing Certificate

This document certifies that the manuscript listed below has been edited to ensure language and grammar accuracy and is error free in these aspects. The logical presentation of ideas and the structure of the paper were also checked during the editing process. The edit was performed by professional editors at Editage, a brand of Cactus Communications. The author's core research ideas were not altered in any way during the editing process. The quality of the edit has been guaranteed, with the assumption that our suggested changes have been accepted and the text has not been further altered without the knowledge of our editors.

MANUSCRIPT TITLE

Risk factors associated with food consumption and food-handling habits for sporadic listeriosis: A case-control study in China from 2013 to 2022

AUTHORS

Yanlin Niu, Weiwei Li, Biyao Xu, Wen Chen, Xiaojuan Qi, Yijing Zhou, Ping Fu, Xiaochen Ma, Yunchang Guo

ISSUED ON

December 15, 2023

JOB CODE

NINLI_8_2

THE EDITAGE Promise QUALITY GUARANTEE

Prabh Grewal
Senior Vice President - Editage

editage | helping you get published

Since 2002, Editage has helped over 430,000 authors publish around 1.2 million research papers in scholarly journals across over 1000 disciplines through editorial, translation, transcription, and publication support services. Editage is a brand of Cactus Communications (cactusglobal.com), a science communication and technology company.

CACTUS.

GLOBAL :
+1(833) 979-0061 | request@editage.com

CHINA :
400-120-3020或021-6020-9400 | fabiao@editage.cn

editage.com | editage.co.kr | editage.jp | editage.cn | editage.com.br | editage.com.tw | editage.de
